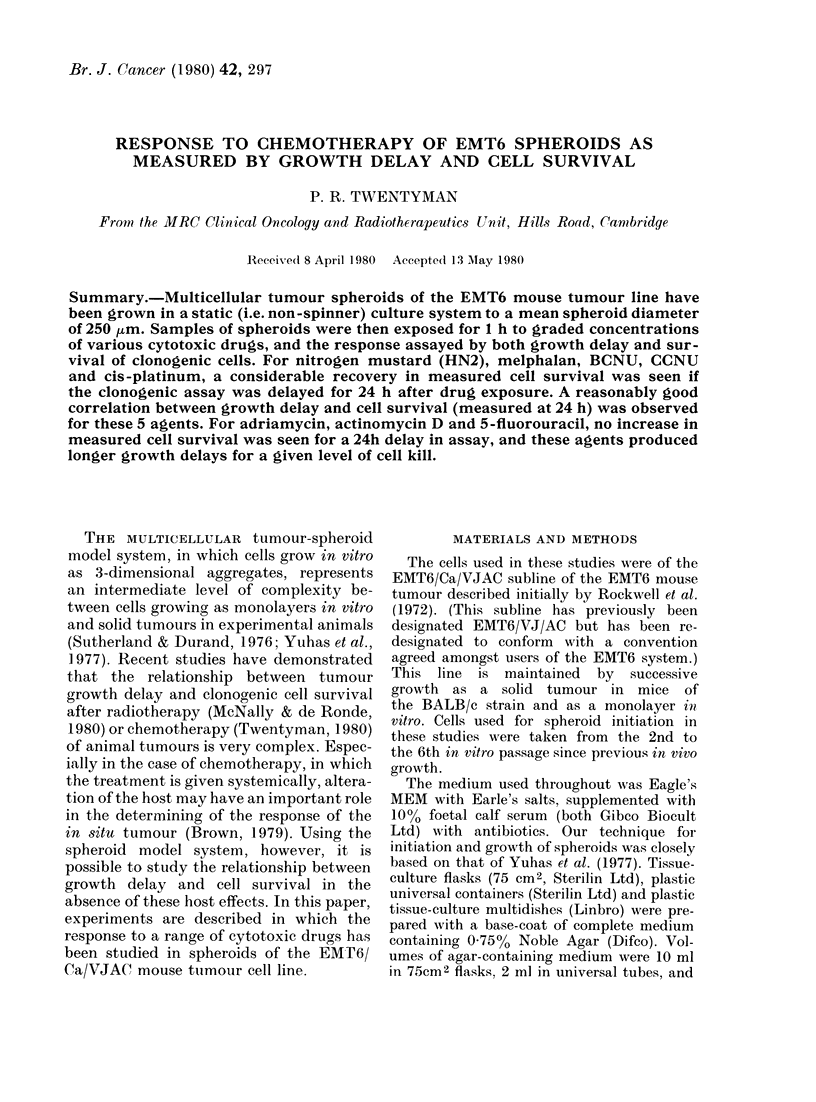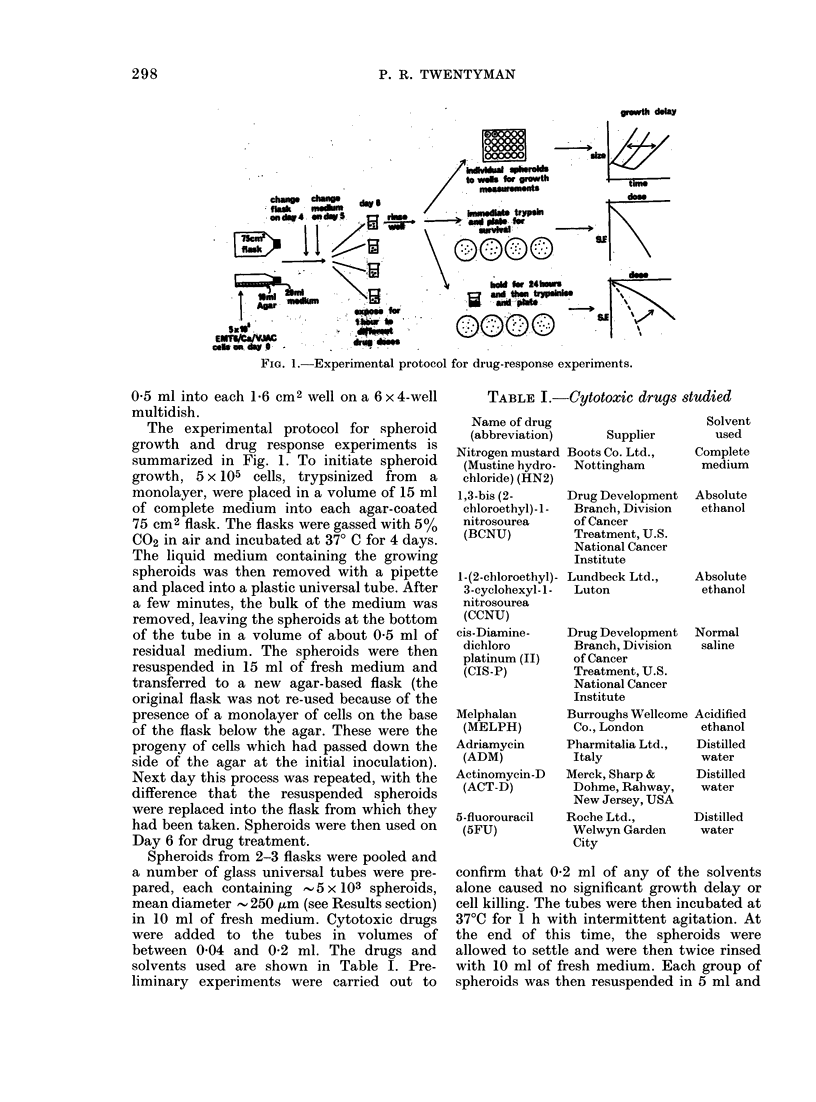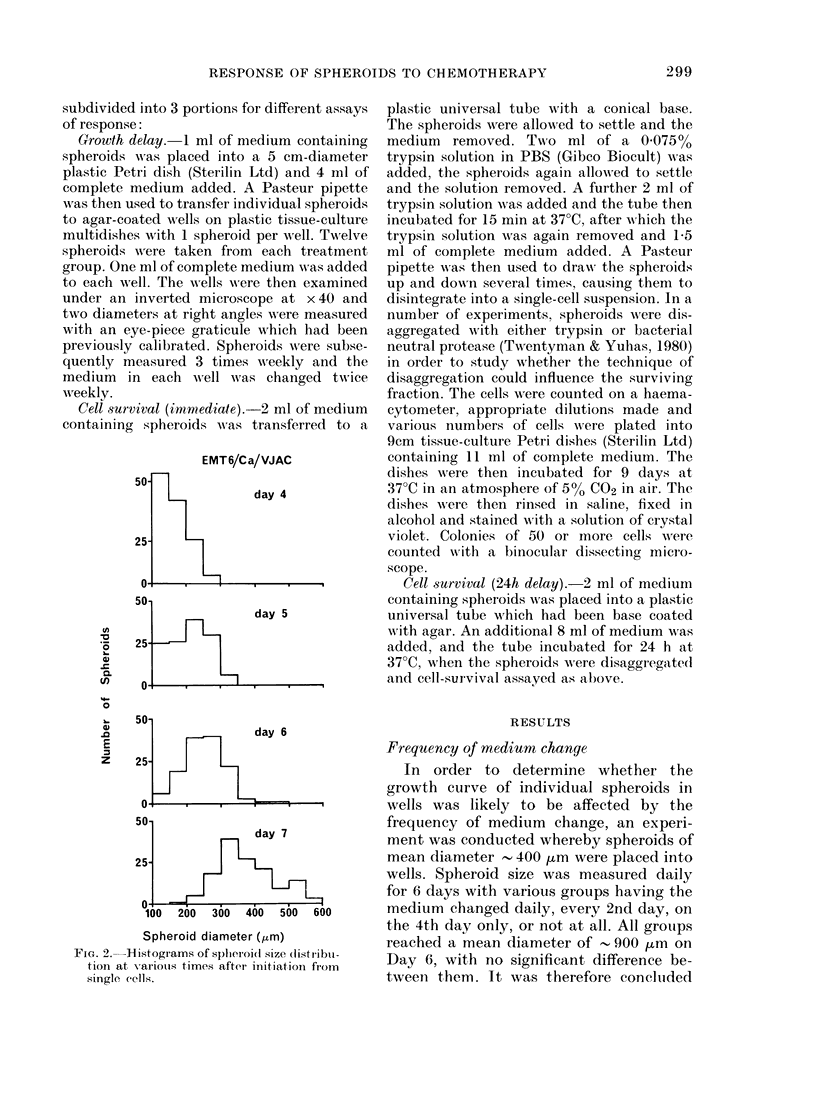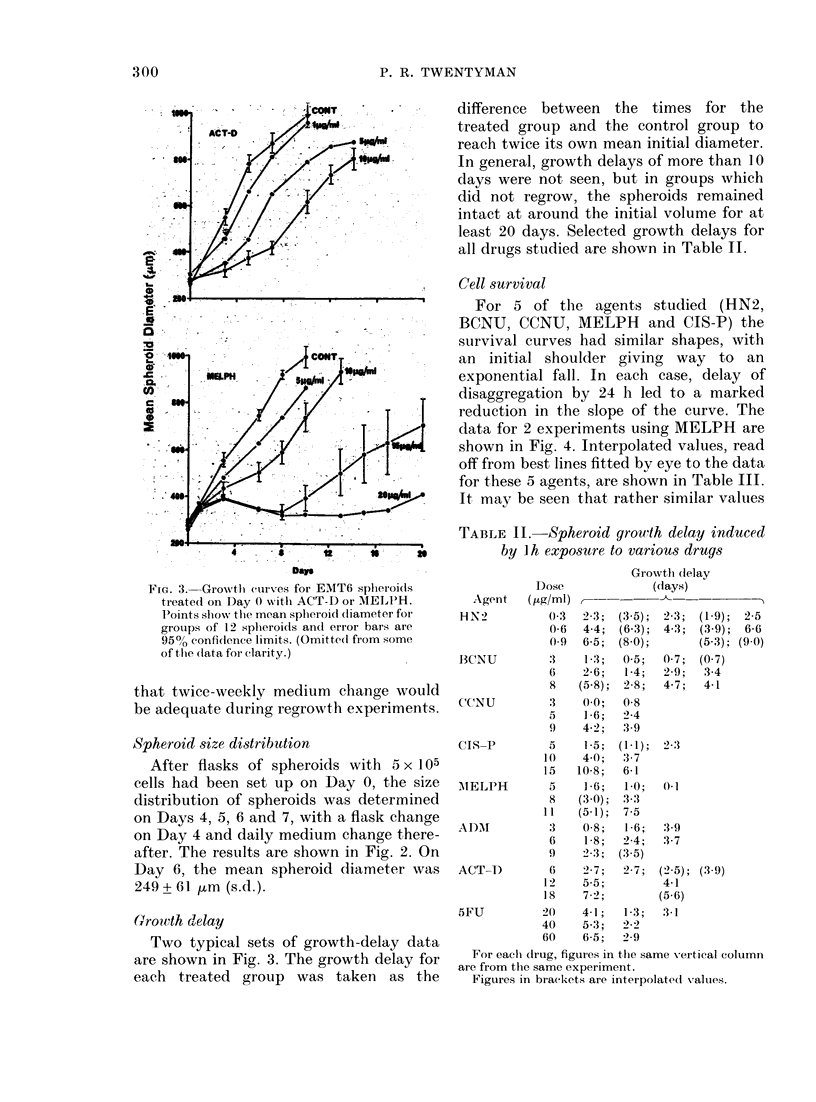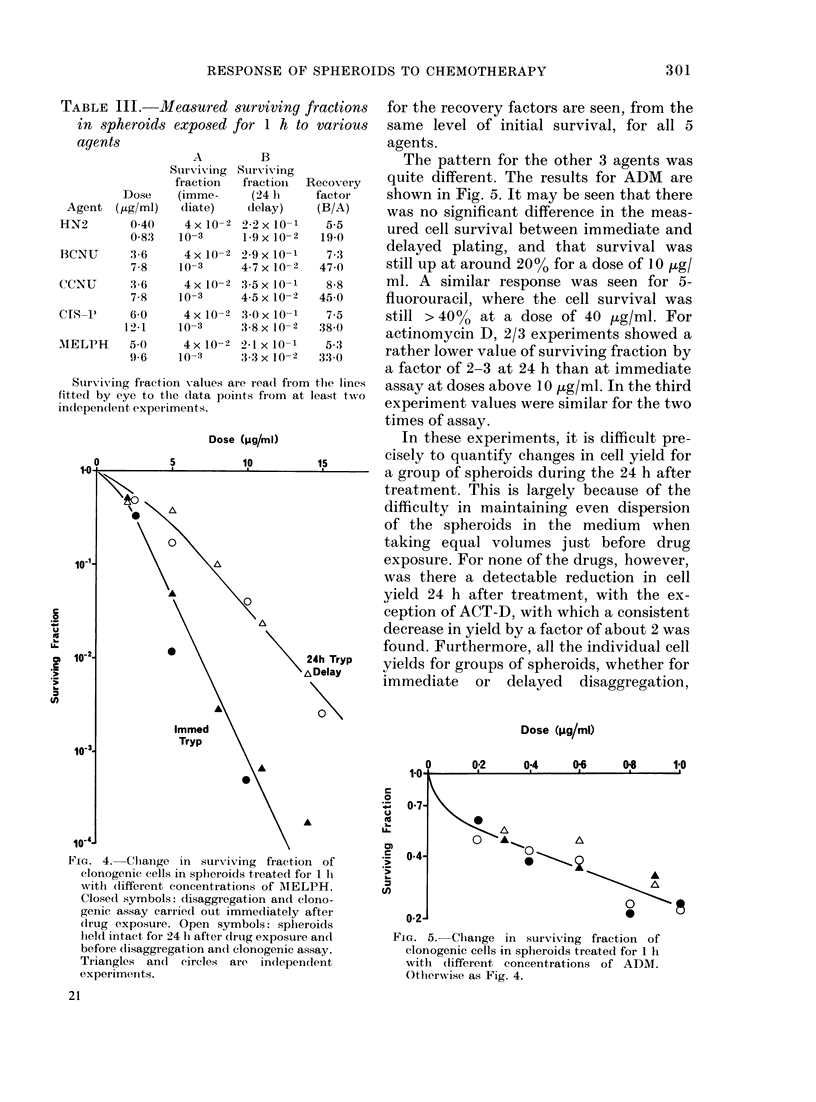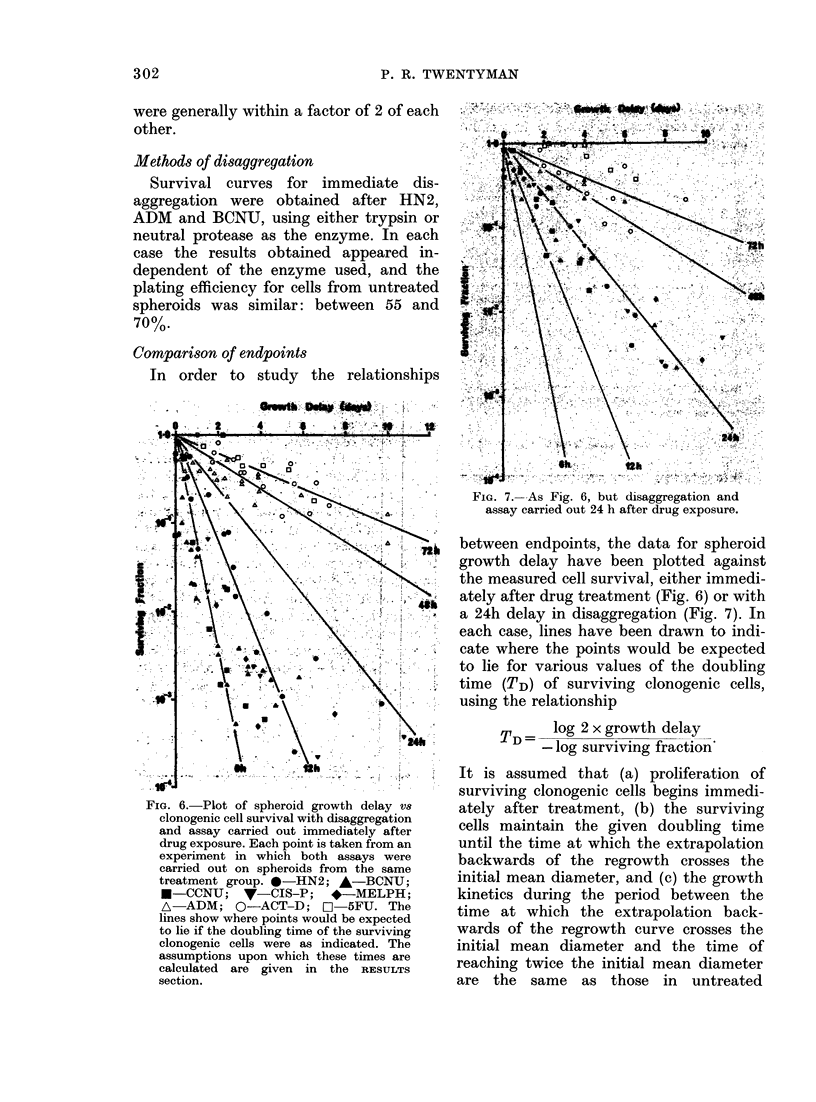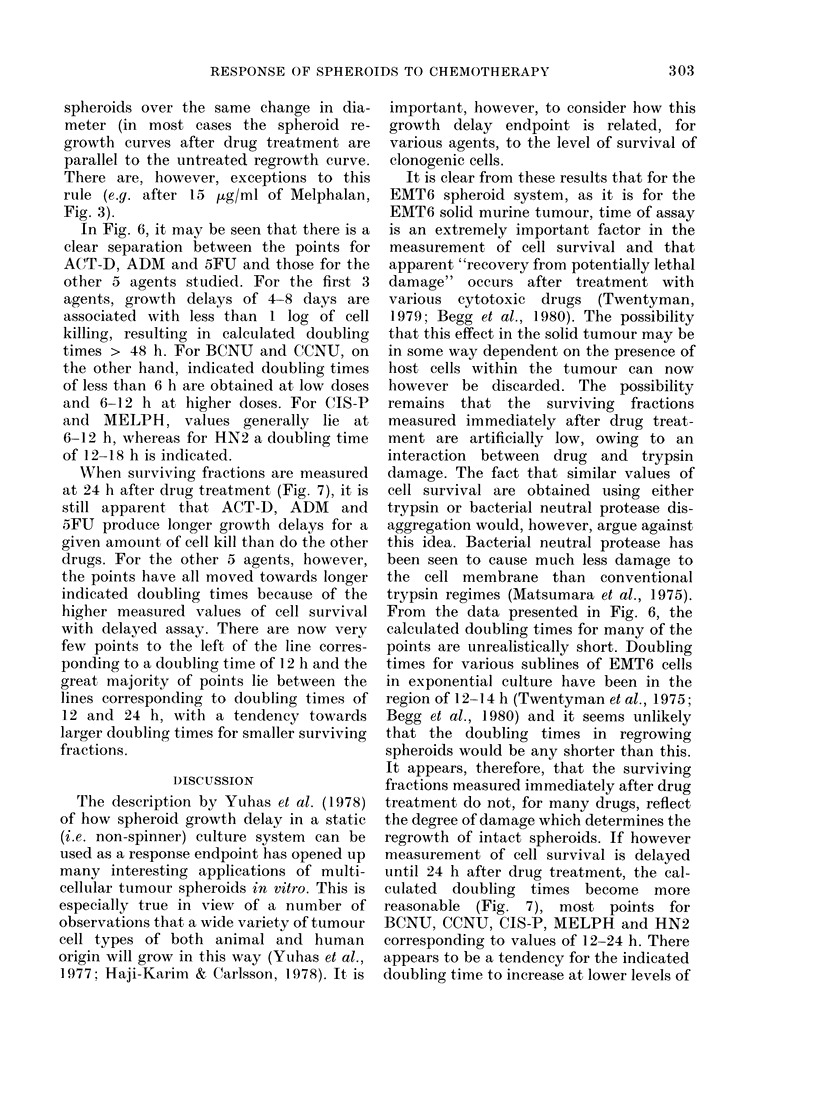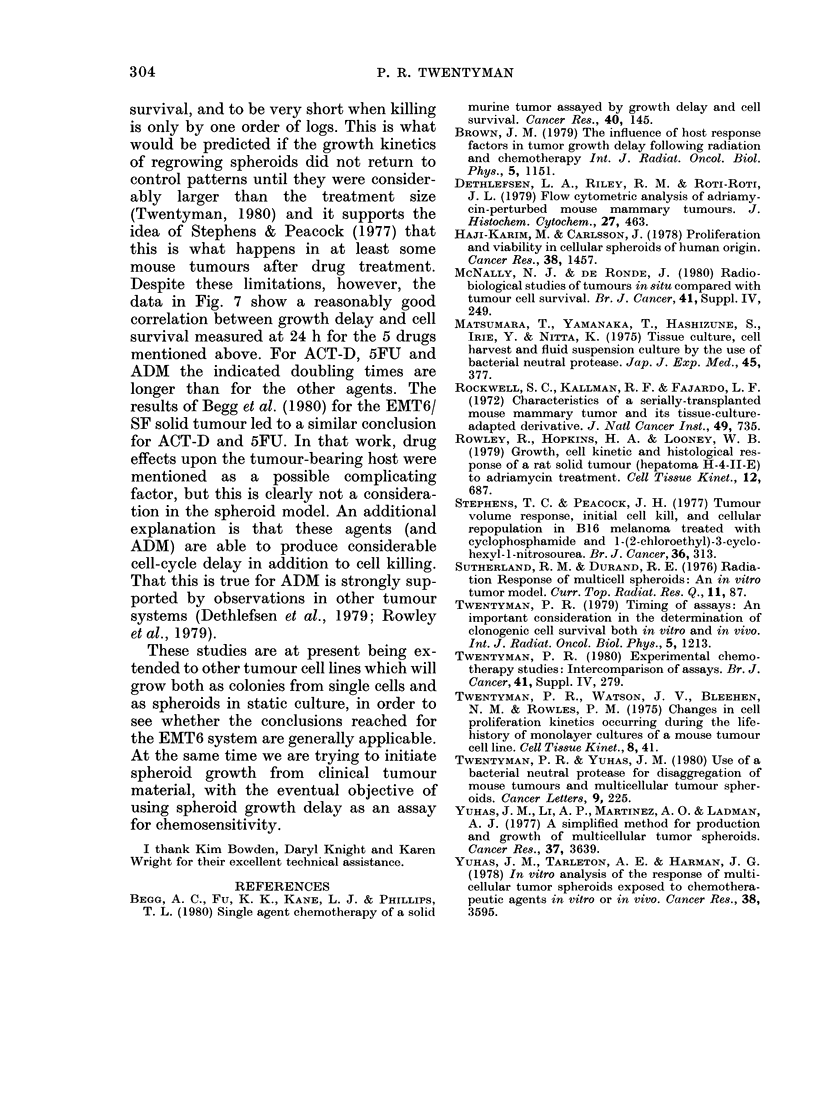# Response to chemotherapy of EMT6 spheroids as measured by growth delay and cell survival.

**DOI:** 10.1038/bjc.1980.230

**Published:** 1980-08

**Authors:** P. R. Twentyman

## Abstract

Multicellular tumour spheroids of the EMT6 mouse tumour line have been grown in a static (i.e. non-spinner) culture system to a mean spheroid diameter of 250 mum. Samples of spheroids were then exposed for 1 h to graded concentrations of various cytotoxic drugs, and the response assayed by both growth delay and survival of clonogenic cells. For nitrogen mustard (HN2), melphalan, BCNU, CCNU and cis-platinum, a considerable recovery in measured cell survival was seen if correlation between growth delay and cell survival (measured at 24 h) was observed for these 5 agents. For adriamycin, actinomycin D and 5-fluorouracil, no increase in measured cell survival was seen for a 24h delay in assay, and these agents produced longer growth delays for a given level of cell kill.


*Br. J. Cancer* (1980) **42**, 297

### RESPONSE TO CHEMOTHERAPY OF EMT6 SPHEROIDS AS MEASURED BY GROWTH DELAY AND CELL SURVIVAL

P. R. TWENTYMAN

*From the MRC Clinical Oncology and Radiotherapeutics Unit, Hills Road, Cambridge*

Received 8 April 1980 Accepted 13 May 1980

**Summary.—Multicellular tumour spheroids of the EMT6 mouse tumour line have been grown in a static (i.e. non-spinner) culture system to a mean spheroid diameter of 250 μm. Samples of spheroids were then exposed for 1 h to graded concentrations of various cytotoxic drugs, and the response assayed by both growth delay and survival of clonogenic cells. For nitrogen mustard (HN2), melphalan, BCNU, CCNU and cis-platinum, a considerable recovery in measured cell survival was seen if the clonogenic assay was delayed for 24 h after drug exposure. A reasonably good correlation between growth delay and cell survival (measured at 24 h) was observed for these 5 agents. For adriamycin, actinomycin D and 5-fluorouracil, no increase in measured cell survival was seen for a 24h delay in assay, and these agents produced longer growth delays for a given level of cell kill.**

THE MULTICELLULAR tumour-spheroid model system, in which cells grow *in vitro* as 3-dimensional aggregates, represents an intermediate level of complexity between cells growing as monolayers *in vitro* and solid tumours in experimental animals (Sutherland & Durand, 1976; Yuhas *et al.*, 1977). Recent studies have demonstrated that the relationship between tumour growth delay and clonogenic cell survival after radiotherapy (McNally & de Ronde, 1980) or chemotherapy (Twentyman, 1980) of animal tumours is very complex. Especially in the case of chemotherapy, in which the treatment is given systemically, alteration of the host may have an important role in the determining of the response of the *in situ* tumour (Brown, 1979). Using the spheroid model system, however, it is possible to study the relationship between growth delay and cell survival in the absence of these host effects. In this paper, experiments are described in which the response to a range of cytotoxic drugs has been studied in spheroids of the EMT6/Ca/VJAC mouse tumour cell line.

### MATERIALS AND METHODS

The cells used in these studies were of the EMT6/Ca/VJAC subline of the EMT6 mouse tumour described initially by Rockwell *et al.* (1972). (This subline has previously been designated EMT6/VJ/AC but has been redesignated to conform with a convention agreed amongst users of the EMT6 system.) This line is maintained by successive growth as a solid tumour in mice of the BALB/c strain and as a monolayer *in vitro*. Cells used for spheroid initiation in these studies were taken from the 2nd to the 6th *in vitro* passage since previous *in vivo* growth.

The medium used throughout was Eagle's MEM with Earle's salts, supplemented with 10% foetal calf serum (both Gibco Biocult Ltd) with antibiotics. Our technique for initiation and growth of spheroids was closely based on that of Yuhas *et al.* (1977). Tissue-culture flasks (75 cm$^2$, Sterilin Ltd), plastic universal containers (Sterilin Ltd) and plastic tissue-culture multidishes (Linbro) were prepared with a base-coat of complete medium containing 0·75% Noble Agar (Difco). Volumes of agar-containing medium were 10 ml in 75cm$^2$ flasks, 2 ml in universal tubes, and

FIG. 1.—Experimental protocol for drug-response experiments.

0·5 ml into each 1·6 $cm^2$ well on a 6 × 4-well multidish.

The experimental protocol for spheroid growth and drug response experiments is summarized in Fig. 1. To initiate spheroid growth, $5 \times 10^5$ cells, trypsinized from a monolayer, were placed in a volume of 15 ml of complete medium into each agar-coated 75 $cm^2$ flask. The flasks were gassed with 5% $CO_2$ in air and incubated at 37° C for 4 days. The liquid medium containing the growing spheroids was then removed with a pipette and placed into a plastic universal tube. After a few minutes, the bulk of the medium was removed, leaving the spheroids at the bottom of the tube in a volume of about 0·5 ml of residual medium. The spheroids were then resuspended in 15 ml of fresh medium and transferred to a new agar-based flask (the original flask was not re-used because of the presence of a monolayer of cells on the base of the flask below the agar. These were the progeny of cells which had passed down the side of the agar at the initial inoculation). Next day this process was repeated, with the difference that the resuspended spheroids were replaced into the flask from which they had been taken. Spheroids were then used on Day 6 for drug treatment.

Spheroids from 2–3 flasks were pooled and a number of glass universal tubes were prepared, each containing $\sim 5 \times 10^3$ spheroids, mean diameter $\sim 250$ μm (see Results section) in 10 ml of fresh medium. Cytotoxic drugs were added to the tubes in volumes of between 0·04 and 0·2 ml. The drugs and solvents used are shown in Table I. Preliminary experiments were carried out to confirm that 0·2 ml of any of the solvents alone caused no significant growth delay or cell killing. The tubes were then incubated at 37°C for 1 h with intermittent agitation. At the end of this time, the spheroids were allowed to settle and were then twice rinsed with 10 ml of fresh medium. Each group of spheroids was then resuspended in 5 ml and

TABLE I.—*Cytotoxic drugs studied*

| Name of drug (abbreviation) | Supplier | Solvent used |
|---|---|---|
| Nitrogen mustard (Mustine hydrochloride) (HN2) | Boots Co. Ltd., Nottingham | Complete medium |
| 1,3-bis (2-chloroethyl)-1-nitrosourea (BCNU) | Drug Development Branch, Division of Cancer Treatment, U.S. National Cancer Institute | Absolute ethanol |
| 1-(2-chloroethyl)-3-cyclohexyl-1-nitrosourea (CCNU) | Lundbeck Ltd., Luton | Absolute ethanol |
| cis-Diamine-dichloro platinum (II) (CIS-P) | Drug Development Branch, Division of Cancer Treatment, U.S. National Cancer Institute | Normal saline |
| Melphalan (MELPH) | Burroughs Wellcome Co., London | Acidified ethanol |
| Adriamycin (ADM) | Pharmitalia Ltd., Italy | Distilled water |
| Actinomycin-D (ACT-D) | Merck, Sharp & Dohme, Rahway, New Jersey, USA | Distilled water |
| 5-fluorouracil (5FU) | Roche Ltd., Welwyn Garden City | Distilled water |

subdivided into 3 portions for different assays of response:

*Growth delay*.—1 ml of medium containing spheroids was placed into a 5 cm-diameter plastic Petri dish (Sterilin Ltd) and 4 ml of complete medium added. A Pasteur pipette was then used to transfer individual spheroids to agar-coated wells on plastic tissue-culture multidishes with 1 spheroid per well. Twelve spheroids were taken from each treatment group. One ml of complete medium was added to each well. The wells were then examined under an inverted microscope at ×40 and two diameters at right angles were measured with an eye-piece graticule which had been previously calibrated. Spheroids were subsequently measured 3 times weekly and the medium in each well was changed twice weekly.

*Cell survival* (*immediate*).—2 ml of medium containing spheroids was transferred to a plastic universal tube with a conical base. The spheroids were allowed to settle and the medium removed. Two ml of a 0·075% trypsin solution in PBS (Gibco Biocult) was added, the spheroids again allowed to settle and the solution removed. A further 2 ml of trypsin solution was added and the tube then incubated for 15 min at 37°C, after which the trypsin solution was again removed and 1·5 ml of complete medium added. A Pasteur pipette was then used to draw the spheroids up and down several times, causing them to disintegrate into a single-cell suspension. In a number of experiments, spheroids were disaggregated with either trypsin or bacterial neutral protease (Twentyman & Yuhas, 1980) in order to study whether the technique of disaggregation could influence the surviving fraction. The cells were counted on a haemacytometer, appropriate dilutions made and various numbers of cells were plated into 9cm tissue-culture Petri dishes (Sterilin Ltd) containing 11 ml of complete medium. The dishes were then incubated for 9 days at 37°C in an atmosphere of 5% $CO_2$ in air. The dishes were then rinsed in saline, fixed in alcohol and stained with a solution of crystal violet. Colonies of 50 or more cells were counted with a binocular dissecting microscope.

*Cell survival* (*24h delay*).—2 ml of medium containing spheroids was placed into a plastic universal tube which had been base coated with agar. An additional 8 ml of medium was added, and the tube incubated for 24 h at 37°C, when the spheroids were disaggregated and cell-survival assayed as above.

FIG. 2.—Histograms of spheroid size distribution at various times after initiation from single cells.

RESULTS

*Frequency of medium change*

In order to determine whether the growth curve of individual spheroids in wells was likely to be affected by the frequency of medium change, an experiment was conducted whereby spheroids of mean diameter ~400 μm were placed into wells. Spheroid size was measured daily for 6 days with various groups having the medium changed daily, every 2nd day, on the 4th day only, or not at all. All groups reached a mean diameter of ~900 μm on Day 6, with no significant difference between them. It was therefore concluded

FIG. 3.—Growth curves for EMT6 spheroids treated on Day 0 with ACT-D or MELPH. Points show the mean spheroid diameter for groups of 12 spheroids and error bars are 95% confidence limits. (Omitted from some of the data for clarity.)

that twice-weekly medium change would be adequate during regrowth experiments.

*Spheroid size distribution*

After flasks of spheroids with $5 \times 10^5$ cells had been set up on Day 0, the size distribution of spheroids was determined on Days 4, 5, 6 and 7, with a flask change on Day 4 and daily medium change thereafter. The results are shown in Fig. 2. On Day 6, the mean spheroid diameter was $249 \pm 61$ $\mu$m (s.d.).

*Growth delay*

Two typical sets of growth-delay data are shown in Fig. 3. The growth delay for each treated group was taken as the difference between the times for the treated group and the control group to reach twice its own mean initial diameter. In general, growth delays of more than 10 days were not seen, but in groups which did not regrow, the spheroids remained intact at around the initial volume for at least 20 days. Selected growth delays for all drugs studied are shown in Table II.

*Cell survival*

For 5 of the agents studied (HN2, BCNU, CCNU, MELPH and CIS-P) the survival curves had similar shapes, with an initial shoulder giving way to an exponential fall. In each case, delay of disaggregation by 24 h led to a marked reduction in the slope of the curve. The data for 2 experiments using MELPH are shown in Fig. 4. Interpolated values, read off from best lines fitted by eye to the data for these 5 agents, are shown in Table III. It may be seen that rather similar values

TABLE II.—*Spheroid growth delay induced by 1h exposure to various drugs*

| Agent | Dose ($\mu$g/ml) | Growth delay (days) | | | | |
|---|---|---|---|---|---|---|
| HN2 | 0·3 | 2·3; | (3·5); | 2·3; | (1·9); | 2·5 |
| | 0·6 | 4·4; | (6·3); | 4·3; | (3·9); | 6·6 |
| | 0·9 | 6·5; | (8·0); | | (5·3); | (9·0) |
| BCNU | 3 | 1·3; | 0·5; | 0·7; | (0·7) | |
| | 6 | 2·6; | 1·4; | 2·9; | 3·4 | |
| | 8 | (5·8); | 2·8; | 4·7; | 4·1 | |
| CCNU | 3 | 0·0; | 0·8 | | | |
| | 5 | 1·6; | 2·4 | | | |
| | 9 | 4·2; | 3·9 | | | |
| CIS-P | 5 | 1·5; | (1·1); | 2·3 | | |
| | 10 | 4·0; | 3·7 | | | |
| | 15 | 10·8; | 6·1 | | | |
| MELPH | 5 | 1·6; | 1·0; | 0·1 | | |
| | 8 | (3·0); | 3·3 | | | |
| | 11 | (5·1); | 7·5 | | | |
| ADM | 3 | 0·8; | 1·6; | 3·9 | | |
| | 6 | 1·8; | 2·4; | 3·7 | | |
| | 9 | 2·3; | (3·5) | | | |
| ACT-D | 6 | 2·7; | 2·7; | (2·5); | (3·9) | |
| | 12 | 5·5; | | 4·1 | | |
| | 18 | 7·2; | | (5·6) | | |
| 5FU | 20 | 4·1; | 1·3; | 3·1 | | |
| | 40 | 5·3; | 2·2 | | | |
| | 60 | 6·5; | 2·9 | | | |

For each drug, figures in the same vertical column are from the same experiment.
Figures in brackets are interpolated values.

TABLE III.—*Measured surviving fractions in spheroids exposed for* 1 *h to various agents*

| Agent | Dose (μg/ml) | A Surviving fraction (immediate) | B Surviving fraction (24 h delay) | Recovery factor (B/A) |
|---|---|---|---|---|
| HN2 | 0·40 | $4\times10^{-2}$ | $2{\cdot}2\times10^{-1}$ | 5·5 |
| | 0·83 | $10^{-3}$ | $1{\cdot}9\times10^{-2}$ | 19·0 |
| BCNU | 3·6 | $4\times10^{-2}$ | $2{\cdot}9\times10^{-1}$ | 7·3 |
| | 7·8 | $10^{-3}$ | $4{\cdot}7\times10^{-2}$ | 47·0 |
| CCNU | 3·6 | $4\times10^{-2}$ | $3{\cdot}5\times10^{-1}$ | 8·8 |
| | 7·8 | $10^{-3}$ | $4{\cdot}5\times10^{-2}$ | 45·0 |
| CIS-P | 6·0 | $4\times10^{-2}$ | $3{\cdot}0\times10^{-1}$ | 7·5 |
| | 12·1 | $10^{-3}$ | $3{\cdot}8\times10^{-2}$ | 38·0 |
| MELPH | 5·0 | $4\times10^{-2}$ | $2{\cdot}1\times10^{-1}$ | 5·3 |
| | 9·6 | $10^{-3}$ | $3{\cdot}3\times10^{-2}$ | 33·0 |

Surviving fraction values are read from the lines fitted by eye to the data points from at least two independent experiments.

FIG. 4.—Change in surviving fraction of clonogenic cells in spheroids treated for 1 h with different concentrations of MELPH. Closed symbols: disaggregation and clonogenic assay carried out immediately after drug exposure. Open symbols: spheroids held intact for 24 h after drug exposure and before disaggregation and clonogenic assay. Triangles and circles are independent experiments.

for the recovery factors are seen, from the same level of initial survival, for all 5 agents.

The pattern for the other 3 agents was quite different. The results for ADM are shown in Fig. 5. It may be seen that there was no significant difference in the measured cell survival between immediate and delayed plating, and that survival was still up at around 20% for a dose of 10 μg/ml. A similar response was seen for 5-fluorouracil, where the cell survival was still >40% at a dose of 40 μg/ml. For actinomycin D, 2/3 experiments showed a rather lower value of surviving fraction by a factor of 2–3 at 24 h than at immediate assay at doses above 10 μg/ml. In the third experiment values were similar for the two times of assay.

In these experiments, it is difficult precisely to quantify changes in cell yield for a group of spheroids during the 24 h after treatment. This is largely because of the difficulty in maintaining even dispersion of the spheroids in the medium when taking equal volumes just before drug exposure. For none of the drugs, however, was there a detectable reduction in cell yield 24 h after treatment, with the exception of ACT-D, with which a consistent decrease in yield by a factor of about 2 was found. Furthermore, all the individual cell yields for groups of spheroids, whether for immediate or delayed disaggregation,

FIG. 5.—Change in surviving fraction of clonogenic cells in spheroids treated for 1 h with different concentrations of ADM. Otherwise as Fig. 4.

were generally within a factor of 2 of each other.

*Methods of disaggregation*

Survival curves for immediate disaggregation were obtained after HN2, ADM and BCNU, using either trypsin or neutral protease as the enzyme. In each case the results obtained appeared independent of the enzyme used, and the plating efficiency for cells from untreated spheroids was similar: between 55 and 70%.

*Comparison of endpoints*

In order to study the relationships between endpoints, the data for spheroid growth delay have been plotted against the measured cell survival, either immediately after drug treatment (Fig. 6) or with a 24h delay in disaggregation (Fig. 7). In each case, lines have been drawn to indicate where the points would be expected to lie for various values of the doubling time ($T_D$) of surviving clonogenic cells, using the relationship

$$T_D = \frac{\log 2 \times \text{growth delay}}{-\log \text{surviving fraction}}.$$

It is assumed that (a) proliferation of surviving clonogenic cells begins immediately after treatment, (b) the surviving cells maintain the given doubling time until the time at which the extrapolation backwards of the regrowth crosses the initial mean diameter, and (c) the growth kinetics during the period between the time at which the extrapolation backwards of the regrowth curve crosses the initial mean diameter and the time of reaching twice the initial mean diameter are the same as those in untreated

FIG. 6.—Plot of spheroid growth delay *vs* clonogenic cell survival with disaggregation and assay carried out immediately after drug exposure. Each point is taken from an experiment in which both assays were carried out on spheroids from the same treatment group. ●—HN2; ▲—BCNU; ■—CCNU; ▼—CIS-P; ◆—MELPH; △—ADM; ○—ACT-D; □—5FU. The lines show where points would be expected to lie if the doubling time of the surviving clonogenic cells were as indicated. The assumptions upon which these times are calculated are given in the RESULTS section.

FIG. 7.—As Fig. 6, but disaggregation and assay carried out 24 h after drug exposure.

spheroids over the same change in diameter (in most cases the spheroid regrowth curves after drug treatment are parallel to the untreated regrowth curve. There are, however, exceptions to this rule (*e.g.* after 15 $\mu$g/ml of Melphalan, Fig. 3).

In Fig. 6, it may be seen that there is a clear separation between the points for ACT-D, ADM and 5FU and those for the other 5 agents studied. For the first 3 agents, growth delays of 4–8 days are associated with less than 1 log of cell killing, resulting in calculated doubling times > 48 h. For BCNU and CCNU, on the other hand, indicated doubling times of less than 6 h are obtained at low doses and 6–12 h at higher doses. For CIS-P and MELPH, values generally lie at 6–12 h, whereas for HN2 a doubling time of 12–18 h is indicated.

When surviving fractions are measured at 24 h after drug treatment (Fig. 7), it is still apparent that ACT-D, ADM and 5FU produce longer growth delays for a given amount of cell kill than do the other drugs. For the other 5 agents, however, the points have all moved towards longer indicated doubling times because of the higher measured values of cell survival with delayed assay. There are now very few points to the left of the line corresponding to a doubling time of 12 h and the great majority of points lie between the lines corresponding to doubling times of 12 and 24 h, with a tendency towards larger doubling times for smaller surviving fractions.

### DISCUSSION

The description by Yuhas *et al.* (1978) of how spheroid growth delay in a static (*i.e.* non-spinner) culture system can be used as a response endpoint has opened up many interesting applications of multicellular tumour spheroids *in vitro*. This is especially true in view of a number of observations that a wide variety of tumour cell types of both animal and human origin will grow in this way (Yuhas *et al.*, 1977; Haji-Karim & Carlsson, 1978). It is important, however, to consider how this growth delay endpoint is related, for various agents, to the level of survival of clonogenic cells.

It is clear from these results that for the EMT6 spheroid system, as it is for the EMT6 solid murine tumour, time of assay is an extremely important factor in the measurement of cell survival and that apparent "recovery from potentially lethal damage" occurs after treatment with various cytotoxic drugs (Twentyman, 1979; Begg *et al.*, 1980). The possibility that this effect in the solid tumour may be in some way dependent on the presence of host cells within the tumour can now however be discarded. The possibility remains that the surviving fractions measured immediately after drug treatment are artificially low, owing to an interaction between drug and trypsin damage. The fact that similar values of cell survival are obtained using either trypsin or bacterial neutral protease disaggregation would, however, argue against this idea. Bacterial neutral protease has been seen to cause much less damage to the cell membrane than conventional trypsin regimes (Matsumara *et al.*, 1975). From the data presented in Fig. 6, the calculated doubling times for many of the points are unrealistically short. Doubling times for various sublines of EMT6 cells in exponential culture have been in the region of 12–14 h (Twentyman *et al.*, 1975; Begg *et al.*, 1980) and it seems unlikely that the doubling times in regrowing spheroids would be any shorter than this. It appears, therefore, that the surviving fractions measured immediately after drug treatment do not, for many drugs, reflect the degree of damage which determines the regrowth of intact spheroids. If however measurement of cell survival is delayed until 24 h after drug treatment, the calculated doubling times become more reasonable (Fig. 7), most points for BCNU, CCNU, CIS-P, MELPH and HN2 corresponding to values of 12–24 h. There appears to be a tendency for the indicated doubling time to increase at lower levels of survival, and to be very short when killing is only by one order of logs. This is what would be predicted if the growth kinetics of regrowing spheroids did not return to control patterns until they were considerably larger than the treatment size (Twentyman, 1980) and it supports the idea of Stephens & Peacock (1977) that this is what happens in at least some mouse tumours after drug treatment. Despite these limitations, however, the data in Fig. 7 show a reasonably good correlation between growth delay and cell survival measured at 24 h for the 5 drugs mentioned above. For ACT-D, 5FU and ADM the indicated doubling times are longer than for the other agents. The results of Begg *et al.* (1980) for the EMT6/SF solid tumour led to a similar conclusion for ACT-D and 5FU. In that work, drug effects upon the tumour-bearing host were mentioned as a possible complicating factor, but this is clearly not a consideration in the spheroid model. An additional explanation is that these agents (and ADM) are able to produce considerable cell-cycle delay in addition to cell killing. That this is true for ADM is strongly supported by observations in other tumour systems (Dethlefsen *et al.*, 1979; Rowley *et al.*, 1979).

These studies are at present being extended to other tumour cell lines which will grow both as colonies from single cells and as spheroids in static culture, in order to see whether the conclusions reached for the EMT6 system are generally applicable. At the same time we are trying to initiate spheroid growth from clinical tumour material, with the eventual objective of using spheroid growth delay as an assay for chemosensitivity.

I thank Kim Bowden, Daryl Knight and Karen Wright for their excellent technical assistance.

### REFERENCES

BEGG, A. C., FU, K. K., KANE, L. J. & PHILLIPS, T. L. (1980) Single agent chemotherapy of a solid murine tumor assayed by growth delay and cell survival. *Cancer Res.*, **40**, 145.

BROWN, J. M. (1979) The influence of host response factors in tumor growth delay following radiation and chemotherapy *Int. J. Radiat. Oncol. Biol. Phys.*, **5**, 1151.

DETHLEFSEN, L. A., RILEY, R. M. & ROTI-ROTI, J. L. (1979) Flow cytometric analysis of adriamycin-perturbed mouse mammary tumours. *J. Histochem. Cytochem.*, **27**, 463.

HAJI-KARIM, M. & CARLSSON, J. (1978) Proliferation and viability in cellular spheroids of human origin. *Cancer Res.*, **38**, 1457.

MCNALLY, N. J. & DE RONDE, J. (1980) Radiobiological studies of tumours *in situ* compared with tumour cell survival. *Br. J. Cancer*, **41**, Suppl. IV, 249.

MATSUMARA, T., YAMANAKA, T., HASHIZUNE, S., IRIE, Y. & NITTA, K. (1975) Tissue culture, cell harvest and fluid suspension culture by the use of bacterial neutral protease. *Jap. J. Exp. Med.*, **45**, 377.

ROCKWELL, S. C., KALLMAN, R. F. & FAJARDO, L. F. (1972) Characteristics of a serially-transplanted mouse mammary tumor and its tissue-culture-adapted derivative. *J. Natl Cancer Inst.*, **49**, 735.

ROWLEY, R., HOPKINS, H. A. & LOONEY, W. B. (1979) Growth, cell kinetic and histological response of a rat solid tumour (hepatoma H-4-II-E) to adriamycin treatment. *Cell Tissue Kinet.*, **12**, 687.

STEPHENS, T. C. & PEACOCK, J. H. (1977) Tumour volume response, initial cell kill, and cellular repopulation in B16 melanoma treated with cyclophosphamide and 1-(2-chloroethyl)-3-cyclohexyl-1-nitrosourea. *Br. J. Cancer*, **36**, 313.

SUTHERLAND, R. M. & DURAND, R. E. (1976) Radiation Response of multicell spheroids: An *in vitro* tumor model. *Curr. Top. Radiat. Res. Q.*, **11**, 87.

TWENTYMAN, P. R. (1979) Timing of assays: An important consideration in the determination of clonogenic cell survival both *in vitro* and *in vivo*. *Int. J. Radiat. Oncol. Biol. Phys.*, **5**, 1213.

TWENTYMAN, P. R. (1980) Experimental chemotherapy studies: Intercomparison of assays. *Br. J. Cancer*, **41**, Suppl. IV, 279.

TWENTYMAN, P. R., WATSON, J. V., BLEEHEN, N. M. & ROWLES, P. M. (1975) Changes in cell proliferation kinetics occurring during the life-history of monolayer cultures of a mouse tumour cell line. *Cell Tissue Kinet.*, **8**, 41.

TWENTYMAN, P. R. & YUHAS, J. M. (1980) Use of a bacterial neutral protease for disaggregation of mouse tumours and multicellular tumour spheroids. *Cancer Letters*, **9**, 225.

YUHAS, J. M., LI, A. P., MARTINEZ, A. O. & LADMAN, A. J. (1977) A simplified method for production and growth of multicellular tumor spheroids. *Cancer Res.*, **37**, 3639.

YUHAS, J. M., TARLETON, A. E. & HARMAN, J. G. (1978) *In vitro* analysis of the response of multicellular tumor spheroids exposed to chemotherapeutic agents *in vitro* or *in vivo*. *Cancer Res.*, **38**, 3595.